# The Treatment of Hysterical Attacks

**Published:** 1887-06

**Authors:** 


**The Treatment of Hysterical Attacks.**—Dr. Albert Ruault gives a simple method which he has found very efficacious in controlling an hysterical fit. It consists in making firm and constant pressure over the supraorbital nerve at its point of emergence from the supraorbital foramen. The head is held securely between the palms of the hands, while pressure is made over the nerve on each side with the thumbs. The writer says that the patients under this treatment first contract the facial muscles with an expression of pain, cry out, and then take several quick successive inspirations. The breath is held for a few seconds, and then, with a long expiration, the muscles relax and the attack is ended. The pressure of the thumb must now be relaxed, otherwise it may have the opposite effect and excite another convulsion. Pressure over any nerve-trunk at the point where it becomes superficial will have the same effect; but the supraorbital nerves are chosen because of their convenient situation.—*France Medicale.*

